# Approximate solutions to several classes of Volterra and Fredholm integral equations using the neural network algorithm based on the sine-cosine basis function and extreme learning machine

**DOI:** 10.3389/fncom.2023.1120516

**Published:** 2023-03-09

**Authors:** Yanfei Lu, Shiqing Zhang, Futian Weng, Hongli Sun

**Affiliations:** ^1^School of Electronics and Information Engineering, Taizhou University, Zhejiang, Taizhou, China; ^2^Data Mining Research Center, Xiamen University, Fujian, Xiamen, China; ^3^School of Mathematics and Statistics, Central South University, Hunan, Changsha, China

**Keywords:** Volterra-Fredholm integral equations, approximate solutions, neural network algorithm, sine-cosine basis function, extreme learning machine

## Abstract

In this study, we investigate a new neural network method to solve Volterra and Fredholm integral equations based on the sine-cosine basis function and extreme learning machine (ELM) algorithm. Considering the ELM algorithm, sine-cosine basis functions, and several classes of integral equations, the improved model is designed. The novel neural network model consists of an input layer, a hidden layer, and an output layer, in which the hidden layer is eliminated by utilizing the sine-cosine basis function. Meanwhile, by using the characteristics of the ELM algorithm that the hidden layer biases and the input weights of the input and hidden layers are fully automatically implemented without iterative tuning, we can greatly reduce the model complexity and improve the calculation speed. Furthermore, the problem of finding network parameters is converted into solving a set of linear equations. One advantage of this method is that not only we can obtain good numerical solutions for the first- and second-kind Volterra integral equations but also we can obtain acceptable solutions for the first- and second-kind Fredholm integral equations and Volterra–Fredholm integral equations. Another advantage is that the improved algorithm provides the approximate solution of several kinds of linear integral equations in closed form (i.e., continuous and differentiable). Thus, we can obtain the solution at any point. Several numerical experiments are performed to solve various types of integral equations for illustrating the reliability and efficiency of the proposed method. Experimental results verify that the proposed method can achieve a very high accuracy and strong generalization ability.

## 1. Introduction

Volterra and Fredholm integral equations have many applications in natural sciences and engineering. A linear phenomenon appearing in many applications in scientific fields can be modeled by linear integral equations (Abdou, 2002; Isaacson and Kirby, 2011). For example, as mentioned by Lima and Buckwar (2015), a class of integro-differential equations, known as neural field equations, describes the large-scale dynamics of spatially structured networks of neurons. These equations are widely used in the field of neuroscience and robotics, and they also play a crucial role in cognitive robotics. The reason is that the architecture of autonomous robots, which are able to interact with other agents in dealing with a mutual task, is strongly inspired by the processing principles and the neuronal circuitry in the primate brain.

This study aims to consider several kinds of linear integral equations. The general form of linear integral equations is defined as follows:


(1) $$\begin{array}{l} \epsilon y\left(x\right)+\lambda \overset{b}{\underset{a}{\int }}k_{1}\left(x,t\right)y\left(t\right)dt+\mu \overset{x}{\underset{a}{\int }}k_{2}\left(x,t\right)y\left(t\right)dt=g\left(x\right),x\in \left[a,b\right], \end{array}$$

Where the functions *k*_1_(*x, t*), *k*_2_(*x, t*), and *g*(*x*) are known, but *y*(*x*) is the unknown function that will be determined; *a* and *b* are constants; and ϵ, λ and μ are parameters. Notably, we have

(i). Equation (1) is called linear Fredholm integral equation of the first kind if ϵ, μ = 0 and λ = 1.
(ii). Equation (1) is called linear Volterra integral equation of the first kind if ϵ, λ = 0 and μ = 1.
(iii). Equation (1) is called linear Fredholm integral equation of the second kind if μ = 0 and ϵ, λ = 1.
(iv). Equation (1) is called linear Volterra integral equation of the second kind if λ = 0 and ϵ, μ = 1.
(v). Equation (1) is called linear Volterra–Fredholm integral equation if μ, ϵ, λ = 1.

Many methods for numerical solutions of Volterra integral equations, Fredholm integral equations, and Volterra-Fredholm integral equations have been presented in recent years. Orthogonal polynomials (e.g., wavelets Maleknejad and Mirzaee, 2005, Bernstein Mandal and Bhattacharya, 2007, Chebyshev Dastjerdi and Ghaini, 2012) were proposed for solving integral equations. The Taylor collocation method (Wang and Wang, 2014), Lagrange collocation method (Wang and Wang, 2013; Nemati, 2015), and Fibonacci collocation method (Mirzaee and Hoseini, 2016) were effective and convenient for solving integral equations. The Sinc-collocation method (Rashidinia and Zarebnia, 2007) and Galerkin method (Saberi-Nadjafi et al., 2012) also give good performance in solving Volterra integral equation problems. However, most of these traditional methods have the following disadvantage: they provide the solution, in the form of an array, at specific preassigned mesh points in the domain, and they need an additional interpolation procedure to yield the solution for the whole domain. In order to have an accurate solution, one either has to increase the order of the method or decrease the step size. This, however, increases the computational cost.

The neural network has excellent application potential in many fields (Habib and Qureshi, 2022; Li and Ying, 2022) owing to its universal function approximation capabilities (Hou and Han, 2012; Hou et al., 2017, 2018). In this case, the neural network is widely used as an effective tool for solving differential equations, integral equations, and integro–differential equations (Mall and Chakraverty, 2014, 2016; Jafarian et al., 2017; Pakdaman et al., 2017; Zuniga-Aguilar et al., 2017; Rostami and Jafarian, 2018). Golbabai and Seifollahi presented radial basis function networks for solving linear Fredholm and Volterra integral equations of the second kind (Golbabai and Seifollahi, 2006), and they solved a system of nonlinear integral equations (Golbabai and Seifollahi, 2009). Effati and Buzhabadia presented multilayer perceptron networks for solving Fredholm integral equations of the second kind (Effati and Buzhabadi, 2012). Jafarian and Nia proposed a feedback neural network method for solving linear Fredholm and Volterra integral equations of the second kind (Jafarian and Nia, 2013a,b). Jafarian presented artificial neural networks-based modeling for solving the Volterra integral equations system (Jafarian et al., 2015). However, the traditional neural network algorithms have some problems, such as over-fitting, difficulty to determine hidden layer nodes, optimization of model parameters, being easily trapped into local minima, slow convergence speed, and reduction in the learning speed and efficiency of the model when the input data are large or the network structure is complex (Huang and Chen, 2008).

Huang et al. (2006a,b) proposed an extreme learning machine (ELM) algorithm, which is a single-hidden-layer feed-forward neural network. The ELM algorithm only needs to set the number of hidden nodes of the network but does not need to adjust the input weights and bias values, and the output weights can be determined by the Moore–Penrose generalized inverse operation. The ELM algorithm provides faster learning speed, better generalization performance, with least human intervention. Based on the advantages, the ELM algorithm has been widely applied to many real-world applications, such as regression and classification problems (Wong et al., 2018). Many neural network methods based on the improved extreme learning machine algorithm for solving ordinary differential equations (Yang et al., 2018; Lu et al., 2022), partial differential equations (Sun et al., 2019; Yang et al., 2020), the ruin probabilities of the classical risk model and the Erlang (2) risk model in Zhou et al. (2019); Lu et al. (2020), and one-dimensional asset-pricing (Ma et al., 2021) have been developed. Chen et al. (2020, 2021, 2022) proposed the trigonometric exponential neural network, Laguerre neural network, and neural finite element method for ruin probability, generalized Black–Scholes differential equation, and generalized Black–Scholes–Merton differential equation. Inspired by these studies, the motivation of this research is to present the sine-cosine ELM (SC-ELM) algorithm to solve linear Volterra integral equations of the first kind, linear Volterra integral equations of the second kind, linear Fredholm integral equations of the first kind, linear Fredholm integral equations of the second kind, and linear Volterra–Fredholm integral equations. In the latest study, a linear integral equation of the third kind with fixed singularities in the kernel is studied by Gabbasov and Galimova (2022), and Volterra integral equations of the first kind on a bounded interval are considered by Bulatov and Markova (2022). For more results, we may refer to Din et al. (2022) and Usta et al. (2022).

In this study, we propose a neural network method based on the sine-cosine basis function and the improved ELM algorithm to solve linear integral equations. Specifically, the hidden layer is eliminated by expanding the input pattern utilizing the sine-cosine basis function, and this simplifies the calculation to some extent. Moreover, the improved ELM algorithm can automatically satisfy the boundary conditions and it transforms the problem into solving a linear system, which provides great convenience for calculation. Furthermore, the closed-form solution by utilizing this model can be obtained, and the approximate solution of any point for linear integral equations can be provided from it.

The remainder of the article is organized as follows. In Section 2, a brief review of the ELM algorithm is provided. In Section 3, a novel neural network method based on the sine-cosine basis function and ELM algorithm for solving integral equations in the form of Equation (1) are discussed. In Section 4, we show several numerical examples to demonstrate the accuracy and the efficiency of the improved neural network algorithm. In Section 5, concluding remarks are presented.

## 2. The ELM algorithm

The ELM algorithm was originated from the single-hidden-layer feed-forward network (SLFN) and then got developed into a generalized SLFN algorithm (Huang and Chen, 2007). The ELM algorithm not only is fully automatically implemented without iterative tuning but also tends to the minimum training error. The ELM algorithm can provide least human intervention, faster learning speed, and better generalization performance. Therefore, the ELM algorithm is widely used in classification and regression tasks (Huang et al., 2012; Cambria and Huang, 2013).

For a data set with *N*+1 different training samples **(*x*_*i*_, *g*_*i*_) ∈ ℝ × ℝ(*i* = 0, 1, ..., *N*)**, the neural network with *M*+1 hidden neurons is expressed as follows:


(2) $$\begin{array}{l} o_{i}=\overset{M}{\underset{j=0}{\sum }}\beta_{j}f\left(w_{j}x_{i}+b_{j}\right),i=0,1,...,N, \end{array}$$

Where *f* is the activation function, *w*_*j*_ is the input weight of the *j*-th hidden layer node, *b*_*j*_ is the bias value of the *j*-th hidden layer node, and β_*j*_ is the output weight connecting the *j*-th hidden layer node and the output node.

The error function of SLFN is as follows:


(3) $$\begin{array}{l} e=\overset{N}{\underset{i=0}{\sum }}||o_{i}-g_{i}||. \end{array}$$

Assuming the error between the output value *o*_*i*_ of SLFN and the exact value *g*_*i*_ is zero, the relationship between *x*_*i*_ and *g*_*i*_ can be modeled as follows:


(4) $$\begin{array}{l} \overset{M}{\underset{j=0}{\sum }}\beta_{j}f\left(w_{j}x_{i}+b_{j}\right)=g_{i},i=0,1,...,N, \end{array}$$

Where both the input weight *w*_*j*_ and the bias value *b*_*j*_ are randomly generated. The equations (4) can be rewritten in the following matrix form, that is:


(5) $$\begin{array}{l} H\beta =G, \end{array}$$

Where *H* is the output matrix of the hidden layer, and it is defined as follows:


$$\begin{array}{lll} H & = & {\left[\begin{array}{cccc} f\left(w_{0}x_{0}+b_{0}\right) & f\left(w_{1}x_{0}+b_{2}\right) & \ldots & f\left(w_{M}x_{0}+b_{M}\right)\\ f\left(w_{0}x_{1}+b_{1}\right) & f\left(w_{1}x_{1}+b_{2}\right) & \ldots & f\left(w_{M}x_{1}+b_{M}\right)\\ \ldots & \ldots & \ddots & \ldots \\ f\left(w_{0}x_{N}+b_{1}\right) & f\left(w_{1}x_{N}+b_{2}\right) & \ldots & f\left(w_{M}x_{N}+b_{M}\right) \end{array}\right]}_{\begin{array}{c} {}_{\left(N+1\right)\times }\\ {}_{\left(M+1\right)} \end{array}},\\ G & = & \left[\begin{array}{c} g_{0}\\ g_{1}\\ \vdots \\ g_{N} \end{array}\right]. \end{array}$$

A common minimum norm least-squares solution of the linear system Equation (5) is calculated by


(6) $$\begin{array}{l} \hat{\beta }=\underset{\beta }{\mathrm{argmin}}||H\beta -G||=H^{†}G. \end{array}$$

## 3. The proposed method

**Figure 1:**
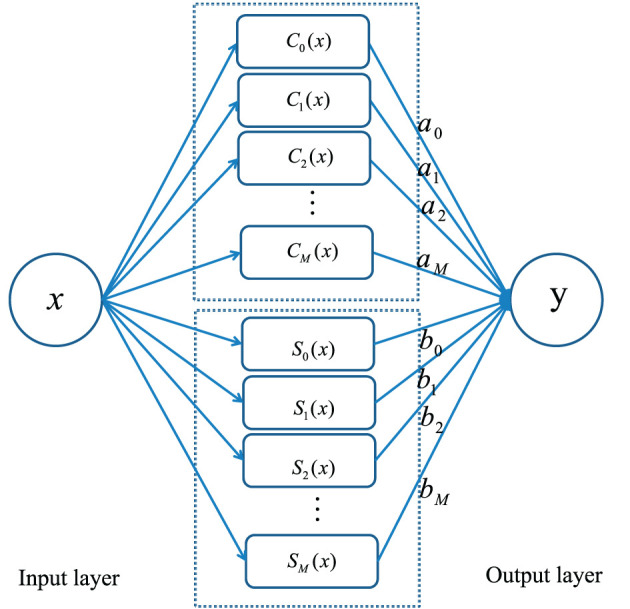
The structure of sine-cosine neural network method for solving several kinds of linear integral equations.

In this section, we propose a neural network method based on sine-cosine basis function and extreme learning machine algorithm to solve linear integral equations. The single-hidden-layer sine-cosine neural network algorithm consists of three layers: an input layer, a hidden layer, and an output layer. The unique hidden layer consists of two parts. The first part uses the cosine basis function as the basis function and the other part implements the superposition of the sine basis function. The structure of sine-cosine neural network method is shown in Figure 1.

The steps of the sine-cosine neural network method for solving several kinds of linear integral equations are as follows:

Step 1: Discretize the interval [*a, b*] into a series of collocation points Ω = {*a* = *x*_0_<*x*_1_ < ... < *x*_*N*_ = *b*}, $x_{i}=a+\frac{b-a}{N}i,i=0,1,...,N$.

Step 2: Construct the approximate solution by using sine-cosine basis as an activation function, that is


(7) $${\hat{y}}_{SC-ELM}(x)=\sum_{j=0}^{M}a_{j}cos(\frac{j\pi }{b-a}(x-a))+\sum_{j=0}^{M}b_{j}sin(\frac{j\pi }{b-a}(x-a)).$$

Step 3: According to different problems and different data sets, we substitute the trial solution ŷ__**SC-ELM**__ into the Equation (1) to be solved. Then, we convert this equation into a matrix form:


(8) $$\begin{array}{l} ||H\hat{\beta }-G||=\underset{\beta }{\min}||H\beta -G||, \end{array}$$

Where $H=\left\{{\left[ha_{ij}\right]}_{N+1,M+1},{\left[hb_{ij}\right]}_{N+1,M+1}\right\};\beta ={\left(a_{0},a_{1},...,a_{M},b_{0},b_{1},...,b_{M}\right)}^{{}^{\prime }};$$ha_{ij}=\epsilon cos\frac{j\pi }{b-a}\left(x_{i}-a\right)+\lambda \overset{b}{\underset{a}{\int }}k_{1}\left(x_{i},t\right)cos\frac{j\pi }{b-a}\left(t-a\right)dt+\mu \overset{x_{i}}{\underset{a}{\int }}k_{2}\left(x_{i},t\right)cos\frac{j\pi }{b-a}\left(t-a\right)dt,i=0,1,...,N,j=0,1,...,M$;$hb_{ij}=\epsilon sin\frac{j\pi }{b-a}\left(x_{i}-a\right)+\lambda \overset{b}{\underset{a}{\int }}k_{1}\left(x_{i},t\right)sin\frac{j\pi }{b-a}\left(t-a\right)dt+\mu \overset{x_{i}}{\underset{a}{\int }}k_{2}\left(x_{i},t\right)sin\frac{j\pi }{b-a}\left(t-a\right)dt,i=0,1,...,N,j=0,1,...,M;$ $G={\left(g\left(x_{0}\right),g\left(x_{1}\right),...,g\left(x_{N}\right)\right)}^{{}^{\prime }}$.

Step 4: From the theory of Moore–Penrose generalized inverse of matrix *H*, we can obtain the net parameters as


(9) $$\begin{array}{l} \hat{\beta }=H^{†}G=\underset{\beta }{\mathrm{argmin}}||H\beta -G||. \end{array}$$

Step 5: Find the connection parameters *a*_*j*_, *b*_*j*_ and the number of neurons *M* with the smallest MSE as the optimal value. The corresponding optimal number of neurons *M* and output weights *a*_*j*_, *b*_*j*_ are, respectively, the optimal number of neurons *M* and optimal output weights $\hat{\beta }$.

Step 6: Substitute *a*_*j*_, *b*_*j*_, *j* = 0, 1, 2, …, *M* into Equation (7) to get the new numerical solution.

Some advantages of the single-layer sine-cosine neural network method for solving integral equations are as follows:

(i) The hidden layer is eliminated by expanding the input pattern using the sine-cosine basis function.
(ii) The sine-cosine neural network algorithm only needs to determine the weights of the output layer. The problem could be transformed into a linear system, and the output weights can be obtained by a simple generalized inverse matrix, which greatly improves the calculation speed.
(iii) We can obtain the closed-form solution by using this model, and most important of all, the approximate solution of any point for linear integral equations can be given from it. It provides a good method for solving integral equations.

## 4. Numerical experiments

In this section, some numerical experiments are performed to demonstrate the reliability and powerfulness of the improved neural network algorithm. The sine-cosine neural network method based on the sine-cosine basis function and ELM algorithm is applied to solve the linear Volterra integral equations of the first kind, linear Volterra integral equations of the second kind, linear Fredholm integral equations of the first kind, linear Fredholm integral equations of the second kind, and linear Volterra–Fredholm integral equations.

The algorithm is evaluated with MATLAB R2021a running in an Intel Xeon Gold 6226R CPU with 64.0GB RAM. The training set is obtained by taking points at equal intervals, and the testing set is randomly selected. The validation set is the set of midpoints *V* = {*v*_*i*_|*v*_*i*_ = (*x*_*i*_+*x*_*i*+1_)/2, *i* = 0, 1, ..., *N*}, where ${\left\{x_{i}\right\}}_{i=0}^{N}$ are training points in the following studies. We use mean square error (MSE), absolute error (AE), mean absolute error (MAE) and root mean square error (RMSE) to measure the error of numerical solution. They can be defined as follows:


(10) $$\begin{array}{l} \begin{array}{c} MSE=\frac{1}{N+1}\overset{N}{\underset{i=0}{\sum }}{\left(y\left(x_{i}\right)-ŷ\left(x_{i}\right)\right)}^{2},\\ AE=|y\left(x_{i}\right)-ŷ\left(x_{i}\right)|,\\ RMSE={\left[\frac{1}{N+1}\overset{N}{\underset{i=0}{\sum }}{\left(y\left(x_{i}\right)-ŷ\left(x_{i}\right)\right)}^{2}\right]}^{\frac{1}{2}}, \end{array} \end{array}$$

Where *y*(*x*_*i*_) denote the exact solution and ŷ(*x*_*i*_) represent the approximate solution obtained by the proposed algorithm. Note that *w*_*j*_ = *jπ*/(*b*−*a*) and *b*_*j*_ = −*jπa*/(*b*−*a*)(*j* = 0, 1, 2, ..., *M*) are selected in our proposed method. Moreover, the number *M* of hidden neurons that results in minimum mean squared error on the validation set can be selected.

### 4.1. Example 1

Consider linear Volterra integral equation of the second kind (Guo et al., 2012) as


(11) $$\begin{array}{l} f\left(x\right)+\overset{x}{\underset{0}{\int }}\left(x-t\right)f\left(t\right)dt=1,x\in \left[0,1\right], \end{array}$$

The analytical solution is *f*(*x*) = *cos*(*x*).

**Figure 2:**
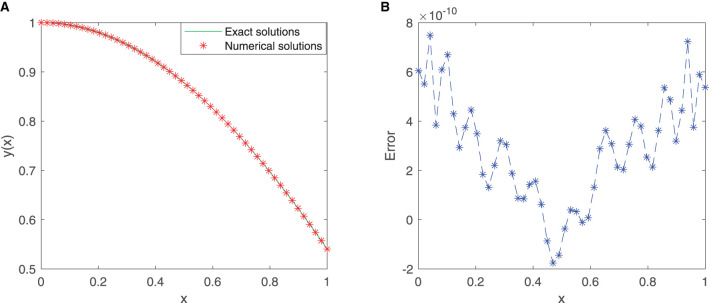
**(A)** Comparison between exact and SC-ELM solutions for Example 1. **(B)** Errors of Example 1.

We train our proposed neural network for 50 equidistant points in the given interval [0, 1] with the first 12 sine-cosine basis functions. Comparison between the exact solution and the approximate solution *via* our improved neural network algorithm is depicted in Figure 2A, and the plot of the error function between them is cited in Figure 2B. As shown in the figures, the mean squared error is 1.3399 × 10^−19^, and the maximum absolute error is approximately 7.4910 × 10^−10^.

Table 1 incorporates the results of the exact solution and the approximate solution *via* our proposed neural network algorithm for 11 testing points at unequal intervals in the domain [0, 1]. The absolute errors are listed in Table 1, in which we observe that the mean squared error is approximately 1.6789 × 10^−19^. These results imply that the proposed method has higher accuracy.

**Table 1:** Comparison between the exact solution and approximate solution (Example 1).

***x***	**Exact solution**	**Approximate solution**	**Absolute error**
0.0624	0.99805375164	0.99805375126	3.8610e-10
0.0915	0.99581679479	0.99581679410	6.9084e-10
0.1518	0.98850048763	0.98850048732	3.1110e-10
0.2410	0.97109978660	0.97109978647	1.2768e-10
0.3604	0.93575583912	0.93575583904	7.4031e-11
0.5252	0.86522368172	0.86522368169	2.7006e-11
0.6395	0.80239425533	0.80239425500	3.2675e-10
0.7590	0.72552456965	0.72552456924	4.1422e-10
0.8482	0.66133438071	0.66133438024	4.7606e-10
0.9084	0.61500816934	0.61500816901	3.3012e-10
0.9348	0.59397933431	0.59397933361	6.9796e-10

Table 2 compares the proposed method with the LS-SVR method. The maximum absolute error is approximately 6.8246 × 10^−10^. Note that in Guo et al. (2012), the maximum absolute error shown in Guo et al. (2012) **Table 5** is approximately 2.4981 × 10^−7^. The solution accuracy of the proposed algorithm is higher.

**Table 2:** Comparison between the SC-ELM method and the LS-SVR method (Example 2).

***x***	**LS-SVR in Guo et al. (2012)**	**SC-ELM**
0.1	7.4597e-08	6.8246e-10
0.2	2.7590e-08	3.7957e-10
0.3	5.1917e-09	3.2404e-10
0.4	2.3898e-07	1.6271e-10
0.5	2.4981e-07	9.4236e-11
0.6	3.8031e-08	4.6072e-11
0.7	2.3423e-07	1.9703e-10
0.8	5.2083e-08	2.3283e-10
0.9	2.4366e-07	3.1284e-10

### 4.2. Example 2

Consider the linear Volterra integral equation of the first kind (Masouri et al., 2010) as


(12) $$\begin{array}{l} \overset{x}{\underset{0}{\int }}e^{x+t}f\left(t\right)dt=xe^{x},x\in \left[0,1\right]. \end{array}$$

The analytical solution is *f*(*x*) = *e*^−*x*^.

**Figure 3:**
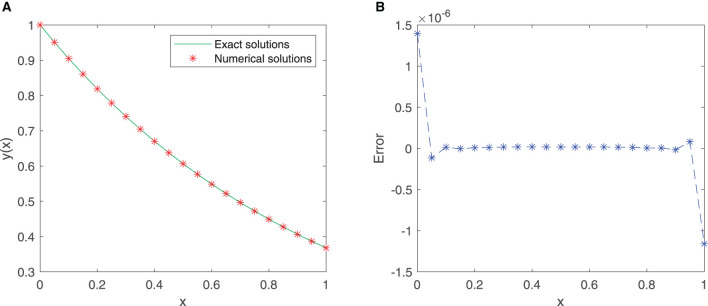
**(A)** Comparison between exact and SC-ELM solutions for Example 2. **(B)** Errors of Example 2.

A total of 21 equidistant points in the given interval [0, 1] are used as the training points, and the neural network adapts the first 10 sine-cosine basis functions. Figures 3A, B shows that the exact solution and the approximate solution are highly consistent. The maximum absolute error is approximately 1.3959 × 10^−6^.

Table 3 lists the results of the exact solution and the approximate solution *via* our proposed neural network algorithm in the domain [0, 1]. The mean squared error is approximately 2.5781 × 10^−16^. These findings provide a strong support for the effectiveness of our proposed method.

**Table 3:** Comparison between exact solution and approximate solution (Example 2).

***x***	**Exact solution**	**Approximate solution**	**Absolute error**
0.0624	0.93950700882	0.93950703293	2.4118e–08
0.0915	0.91256131615	0.91256129442	2.1728e–08
0.1518	0.85916009558	0.85916009819	2.6073e–09
0.2410	0.78584162639	0.78584161623	1.0154e–08
0.3604	0.69739731135	0.69739729287	1.8481e–08
0.5252	0.59143706512	0.59143704802	1.7099e–08
0.6395	0.52755613618	0.52755611864	1.7535e–08
0.7590	0.46813432735	0.46813431582	1.1524e–08
0.8482	0.42818497165	0.42818496663	5.0275e–09
0.9084	0.40316877830	0.40316880212	2.3823e–08
0.9348	0.39266439056	0.39266439284	2.2803e–09

### 4.3. Example 3

We consider linear Fredholm integral equation of the first kind (Rashed, 2003) as


(13) $$\begin{array}{l} \overset{1}{\underset{0}{\int }}{\left(x^{2}+t^{2}\right)}^{\frac{1}{2}}f\left(t\right)dt=\frac{{\left(1+x^{2}\right)}^{\frac{3}{2}}-x^{3}}{3}. \end{array}$$

The analytical solution is *f*(*x*) = *x*.

**Figure 4:**
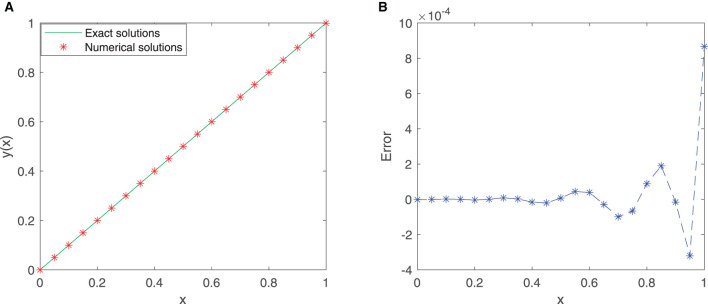
**(A)** Comparison between exact and SC-ELM solutions for Example 3. **(B)** Errors of Example 3.

This problem is solved by utilizing our proposed neural network model in the given interval [0, 1]. We consider 21 equidistant points in the domain [0, 1] with the first six sine-cosine basis functions to train the model. Comparison between the exact solution and the approximate solution *via* our improved neural network algorithm is depicted in Figure 4A, and the error plot is depicted in Figure 4B. Note that the mean squared error is 4.5915 × 10^−8^ for these training points.

Table 4 incorporates the results of the exact solution and the approximate solution *via* our proposed neural network algorithm for 11 testing points at unequal intervals in the domain [0, 1]. We observe that the maximum absolute error is approximately 2.7433 × 10^−4^. The results show that this new neural network has a good generalization ability.

**Table 4:** Comparison between the exact solution and approximate solution (Example 3).

***x***	**Exact solution**	**Approximate solution**	**Absolute error**
0.0624	0.0624	0.0624007691887	7.6919e–07
0.0915	0.0915	0.0914990435138	9.5649e–07
0.1518	0.1518	0.1517998073669	1.9263e–07
0.2410	0.2410	0.2410008795702	8.7957e–07
0.3604	0.3604	0.3604013108547	1.3109e–06
0.5252	0.5252	0.5251722711564	2.7729e–05
0.6395	0.6395	0.6395114159462	1.1416e–05
0.7590	0.7590	0.7590451800564	4.5180e–05
0.8482	0.8482	0.8480097658921	1.9023e–04
0.9084	0.9084	0.9084802272880	8.0227e–05
0.9348	0.9348	0.9350743334136	2.7433e–04

### 4.4. Example 4

We consider the linear Fredholm integral equation of the second kind (Golbabai and Seifollahi, 2006) as


(14) $$\begin{array}{l} f\left(x\right)+\frac{1}{3}\overset{1}{\underset{0}{\int }}e^{2x-\frac{5t}{3}}f\left(t\right)dt=e^{2x+\frac{1}{3}}. \end{array}$$

The analytical solution is *f*(*x*) = *e*^2*x*^.

**Figure 5:**
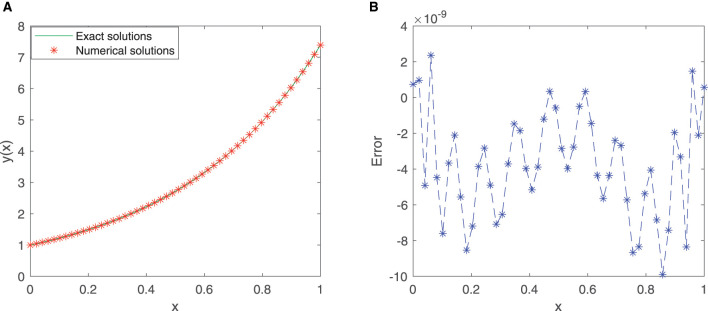
**(A)** Comparison between exact and SC-ELM solutions for Example 4. **(B)** Errors of Example 4.

The improved neural network algorithm for the linear Fredholm integral equation of the second kind has been trained with 50 equidistant points in the given interval [0, 1] with the first 12 sine-cosine basis functions. The approximate solution obtained by the improved neural network algorithm and the exact solution are shown in Figure 5A, and the error function is displayed in Figure 5B. Especially, the mean squared error is 2.3111 × 10^−17^, and the maximum absolute error is approximately 9.8998 × 10^−9^, which fully demonstrates the superiority of the improved neural network algorithm.

Finally, Table 5 provides the results of the exact solution and the approximate solution *via* our proposed neural network algorithm for 11 testing points at unequal intervals in the domain [0, 1]. As shown in Table 5, the mean squared error is approximately 3.1391 × 10^−17^, which undoubtedly shows the power and effectiveness of the proposed method.

**Table 5:** Comparison between the exact solution and approximate solution (Example 4).

***x***	**Exact solution**	**Approximate solution**	**Absolute error**
0.0624	1.13292184603767	1.13292184381566	2.2220e–09
0.0915	1.20081440808083	1.20081441530638	7.2255e–09
0.1518	1.35472705687431	1.35472706011551	3.2412e–09
0.2410	1.61930978530193	1.61930978804438	2.7425e–09
0.3604	2.05607741480638	2.05607741623227	1.4259e–09
0.5252	2.85879440715296	2.85879441106042	3.9075e–09
0.6395	3.59304488356426	3.59304488863783	5.0735e–09
0.7590	4.56308988310901	4.56308989202265	8.9136e–09
0.8482	5.45427660976895	5.45427661875143	8.9825e–09
0.9084	6.15214006907956	6.15214007048970	1.4101e–09
0.9348	6.48570159972151	6.48570160778124	8.0597e–09

Table 6 compares the proposed method with RBF networks. The maxmium absolute error by our proposed method is approximately 7.7601 × 10^−9^. Note that in Golbabai and Seifollahi (2006), the maxmium absolute error shown in Golbabai and Seifollahi (2006), as shown in Table 1, is approximately 6.7698 × 10^−7^. The solution accuracy of the proposed algorithm is higher.

**Table 6:** Comparison between the SC-ELM method and RBF method (Example 4).

***x***	**RBF in Golbabai and Seifollahi (2006)**	**SC-ELM**
0.1	4.1721e-07	7.7331e-09
0.2	1.6226e-07	7.7601e-09
0.3	9.9728e-08	7.0314e-09
0.4	5.3328e-07	4.9446e-09
0.5	5.1282e-07	1.7010e-09
0.6	8.8658e-08	9.5548e-11
0.7	3.8239e-07	2.1508e-09
0.8	6.7698e-07	4.8329e-09
0.9	3.3687e-07	1.6513e-09

### 4.5. Example 5

Consider the linear Volterra–Fredholm integral equation (Wang and Wang, 2014) as


(15) $$\begin{array}{l} y\left(x\right)+\overset{1}{\underset{0}{\int }}e^{x+t}y\left(t\right)dt-\overset{x}{\underset{0}{\int }}e^{x+t}y\left(t\right)dt=e^{-x}-e^{x}\left(x-1\right). \end{array}$$

The analytical solution is *f*(*x*) = *e*^−*x*^.

**Figure 6:**
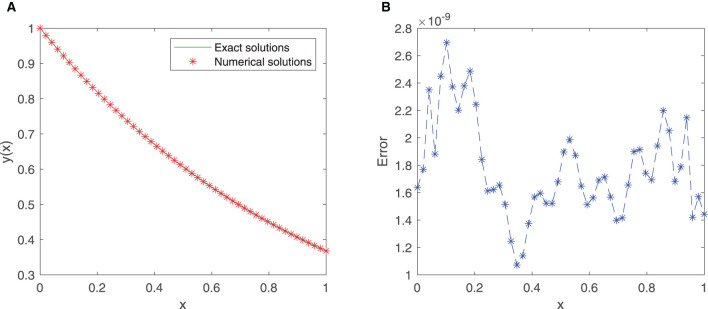
**(A)** Comparison between exact and SC-ELM solutions for Example 5. **(B)** Errors of Example 5.

A total of 50 equidistant points in the given interval [0, 1] and the first 11 sine-cosine basis functions are considered to train the neural network model. The comparison images and error images of the exact solution and the approximate solution are listed in Figures 6A, B, from which we can see that the mean squared error is 3.3499 × 10^−18^.

Table 7 shows the results of the exact solution and the approximate solution *via* the improved ELM method for 11 testing points at unequal intervals in the domain [0, 1]. As shown in the table, the maximum absolute error is approximately 2.6673 × 10^−9^, which reveals that the improved neural network algorithm has higher accuracy and excellent performance.

**Table 7:** Comparison between the exact solution and approximate solution (Example 5).

***x***	**Exact solution**	**Approximate solution**	**Absolute error**
0.0624	0.93950700882	0.93950700692	1.8957e-09
0.0915	0.91256131615	0.91256131348	2.6673e-09
0.1518	0.85916009558	0.85916009332	2.2550e-09
0.2410	0.78584162639	0.78584162475	1.6352e-09
0.3604	0.69739731135	0.69739731026	1.0886e-09
0.5252	0.59143706512	0.59143706314	1.9856e-09
0.6395	0.52755613618	0.52755613446	1.7191e-09
0.7590	0.46813432735	0.46813432542	1.9239e-09
0.8482	0.42818497165	0.42818496954	2.1172e-09
0.9084	0.40316877830	0.40316877665	1.6478e-09
0.9348	0.39266439056	0.39266438843	2.1275e-09

We compare the RMSE of our proposed method and the Taylor collocation method in Wang and Wang (2014). From Table 8, we can see clearly that our algorithm is more accurate than the algorithm in the Taylor collocation method. As can be seen from Table 8, when 5, 8, and 9 points are tested, the RMSEs shown by the Taylor collocation method in Wang and Wang (2014) are approximately 4.03 × 10^−7^, 9.50 × 10^−7^, and 2.15 × 10^−5^, but the RMSEs shown by our proposed method are respectively 1.67 × 10^−9^, 1.78 × 10^−9^, and 1.67 × 10^−9^.

**Table 8:** RMSE comparison of Example 5.

***N***	**Taylor solution**	**SC-ELM solution**
5	4.03e-07	1.67e-09
8	9.50e-07	1.78e-09
9	2.15e-05	1.67e-09

### 4.6. Example 6

We consider linear the Volterra integral equation of the second kind (Saberi-Nadjafi et al., 2012).


(16) $$\begin{array}{l} f(x)+\int_{0}^{x}\left(8-2x^{2}\right)sin(xt)f(t)dt=-2x+4sin(x^{2})(sin2x\\ \;-cos2x)+(sin2x+cos2x)(1+2xcos(x^{2})). \end{array}$$

The analytical solution is *f*(*x*) = *sin*(2*x*)+*cos*(2*x*).

**Figure 7:**
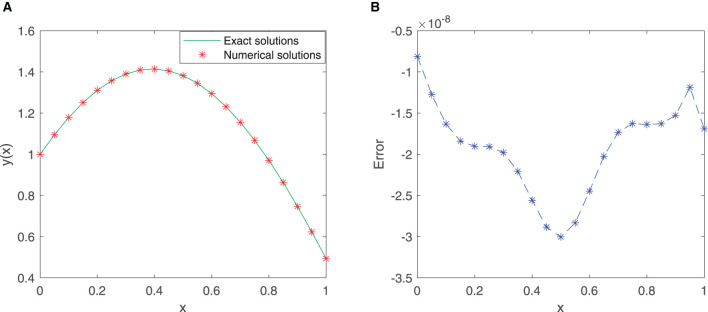
**(A)** Comparison between exact and SC-ELM solutions for Example 6. **(B)** Errors of Example 6.

A total of 21 equidistant discrete points and the first 11 sine-cosine basis functions are utilized to construct the neural network model. The comparison images and error images of the exact solution and the approximate solution are displayed in Figures 7A, B. It ia not hard to find that the MSE is 4.2000 × 10^−16^, and this implies that the proposed algorithm has higher accuracy.

To verify the effectiveness of our proposed method, we provide the results of the exact solution and the approximate solution *via* the improved ELM method for 11 testing points at unequal intervals in the domain [0, 1], see Table 9. As shown in the table, the maximum absolute error is approximately 2.9539 × 10^−8^, which shows that the proposed algorithm has good generalization ability.

**Table 9:** Comparison between the exact solution and approximate solution (Example 6).

***x***	**Exact solution**	**Approximate solution**	**Absolute error**
0.0624	1.1166988736914	1.1166988834032	9.7117e-09
0.0915	1.1652824720246	1.1652824868524	1.4828e-08
0.1518	1.2532239237305	1.2532239421393	1.8409e-08
0.2410	1.3496218317010	1.3496218507822	1.9081e-08
0.3604	1.4112638863693	1.4112639090979	2.2729e-08
0.5252	1.3648462234191	1.3648462529585	2.9539e-08
0.6395	1.2454017480945	1.2454017691674	2.1073e-08
0.7590	1.0513783999948	1.0513784162122	1.6217e-08
0.8482	0.8668485498714	0.8668485662364	1.6365e-08
0.9084	0.7263634857982	0.7263635021492	1.6351e-08
0.9348	0.6613122433253	0.6613122627673	1.9442e-08

Table 10 compares the MSE of the numerical solutions obtained by the SC-ELM model when more training points are added and different numbers of hidden layer neurons are configured. From these results, it can be seen that the proposed method can achieve good accuracy. The calculation time of different examples is listed in Table 11. These data suggest that our method is efficient and feasible.

**Table 10:** Comparison of the different examples of MSE with different numbers of training points and hidden neurons.

**MSE**	***M*** **= 5, *N* = 20**	***M*** **= 10, *N* = 20**	***M*** **= 10, *N* = 100**
Example 1	5.4420e-11	3.6233e-17	6.9420e-19
Example 2	2.7384e-09	1.9056e-12	6.3433e-17
Example 3	4.5915e-08	4.4380e-05	4.8435e-06
Example 4	2.8418e-08	4.5969e-16	3.8451e-16
Example 5	1.6398e-10	8.0501e-17	2.0969e-18
Example 6	2.8025e-11	4.2000e-16	4.0548e-19

**Table 11:** Execution time of different examples.

**Example**	***t***
Example 1	0.3317
Example 2	0.1505
Example 3	0.1080
Example 4	0.3391
Example 5	0.6356
Example 6	0.1683

## 5. Conclusion

In this study, the improved neural network algorithm based on the sine-cosine basis function and extreme learning machine algorithm has been developed for solving linear integral equations. The accuracy of the improved neural network has been checked by solving a linear Volterra integral equation of the first kind, a linear Volterra integral equation of the second kind, a linear Fredholm integral equation of the first kind, a linear Fredholm integral equation of the second kind, and a linear Volterra-Fredholm integral equation. The experimental results of the improved ELM approach with different types of integral equations show that the simulation results are close to the exact results. Therefore, the proposed model is very precise and could be a good tool for solving linear integral equations.

## Data availability statement

The original contributions presented in the study are included in the article/supplementary material, further inquiries can be directed to the corresponding author.

## Author contributions

All authors listed have made a substantial, direct, and intellectual contribution to the work and approved it for publication.
